# Prevalence of sarcopenia in Africa: a systematic review and meta-analysis of observational studies

**DOI:** 10.1007/s40520-023-02671-w

**Published:** 2024-01-28

**Authors:** Nicola Veronese, Lee Smith, Ai Koyanagi, Jaco Hoffman, Mouna Snoussi, Konstantinos Prokopidis, Ligia J. Dominguez, Mario Barbagallo

**Affiliations:** 1 Department of Internal Medicine, Geriatrics Section, University of Palermo, Palermo, Italy; 2 Centre for Health Performance and Wellbeing, Anglia Ruskin University, Cambridge, UK; 3 Research and Development Unit, Parc Sanitari Sant Joan de Déu, Sant Boi de Llobregat, Barcelona, Spain; 4 Optentia Research Unit, North-West University, Vanderbijlpark, South Africa; 5 Department of Internal Medicine, Hedi Chaker Hospital, Medical School of Sfax, Sfax, Tunisia; 6 Department of Musculoskeletal and Ageing Science, Institute of Life Course and Medical Sciences, University of Liverpool, Liverpool, UK; 7 Faculty of Medicine and Surgery, University Kore of Enna, 94100 Enna, Italy

**Keywords:** Sarcopenia, Africa, Prevalence, Epidemiology

## Abstract

**Background:**

Existing literature suggests that sarcopenia is a highly prevalent condition in older people. However, most studies to date reporting data on its prevalence have been mainly carried out in Western countries, while data on sarcopenia in Africa is scarce. With this systematic review and meta‐analysis, we aimed to determine the prevalence of sarcopenia in African countries and to explore potential factors that could explain higher or lower prevalence of this condition in Africa.

**Methods:**

Major databases for studies reporting data on sarcopenia in African countries were searched from inception to June 2023. We conducted a meta-analysis of the prevalence [and 95% confidence intervals (95% CIs)] of sarcopenia in Africa, applying a random effect model. Several sensitivity and meta-regression analyses were run.

**Results:**

Among 147 articles initially screened, six articles (with seven cohorts) including a total of 10,656 participants were included. Mean age of participants was 66.9 years, and the majority were female (58.1%). The weighted prevalence of sarcopenia in the selected countries of Africa was 25.72% (95%CI: 18.90–32.55). This outcome was characterized by a high heterogeneity (*I*^2^ = 99%) and by publication bias. Among the factors investigated, sarcopenia was lower when assessed using only one anthropometric measure, or in South Africa.

**Conclusion:**

Sarcopenia is a prevalent condition in Africa and thus research regarding this topic is a public health priority. Future studies that cover African countries for which data are not available and using standardized criteria are needed.

**Supplementary Information:**

The online version contains supplementary material available at 10.1007/s40520-023-02671-w.

## Introduction

Sarcopenia usually refers to the pathological loss of quantity and quality of skeletal muscle mass that leads to a loss in muscle strength and physical performance [1]. Sarcopenia is associated with several negative outcomes among older people [2], and recently has been recognized as a geriatric syndrome [3]. Over more than three decades, sarcopenia has been recognized as a condition of clinical importance, and is now included in the International Classification of Disease [4].

Sarcopenia is known to be highly prevalent in older people [5], but increasing research is showing its importance in specialties other than geriatrics, such as cardiology [6] or oncology [7]. The pathophysiology of sarcopenia is extremely complex and may result from biological alterations in the structure of the muscles, endocrinological issues, and malnutrition [8]. Despite the importance of sarcopenia, a single diagnostic criterion has not yet been established, and nowadays, several societies have proposed a combination of low muscle mass, muscle strength, and physical performance using different cut-offs and criteria [9–11]. As expected, the different criteria used have led to a very heterogeneous prevalence of sarcopenia. Some previous systematic reviews and meta‐analyses reported the prevalence only in relatively healthy older adults [5], or only among community‐dwelling older people [12]. A more recent systematic review including all settings and several countries reported an overall prevalence of sarcopenia between 10% and 27% [13].

However, a potential limitation of these meta-analyses is that the prevalence of sarcopenia in Africa was poorly explored. This is an important research gap as several geriatric syndromes are dramatically increasing in this continent. For example, it is estimated that the overall prevalence of dementia among older adults in Africa is approximately 2.4% [14] and in the coming years, dementia will reach similar figures to those of Western countries [15]. Other data indicate that the prevalence of geriatric syndromes is high in Africa. For example, in a cross-sectional study carried out in Cameroon, it was found that these conditions may affect up to two-thirds of older people [16].

Given this background, with this systematic review and meta‐analysis, we aimed to determine the prevalence of sarcopenia in African countries and to explore potential factors that could explain higher or lower prevalence of this condition.

## Materials and methods

### Protocol registration

This study was conducted following the recommendations in the Cochrane handbook for systematic literature reviews [17]. This systematic review and meta-analysis was reported following the Preferred Reporting Items for Systematic Reviews and Meta-Analyses (PRISMA) guidelines, updated version to 2020 [18]. The protocol was registered in OSF (https://osf.io/pnxh7/).

### Information sources and search strategies

The research question for this systematic review is: “What is the prevalence of sarcopenia in Africa?” We searched Medline (via Ovid), Embase, and Web of Science from database inception to 01st June 2023. The search for individual studies in these bibliographic databases was supplemented by a manual search of reference lists included in identified articles.

We built the following search strategy for Medline: “(Africa OR Angola OR Algeria OR Benin OR Botswana OR Burkina Faso OR Burundi OR Cameroon OR Cape Verde OR Chad OR Central African Republic OR Ciad OR Comore OR Ivory Cost OR Congo OR Egypt OR Eritrea OR Ethiopia OR Gabon OR Gambia OR Ghana OR Djibuti OR Guinea OR Kenya OR Lesotho OR Liberia OR Libya OR Madagascar OR Malawi OR Mali OR Mauritania OR Mauritius OR Morocco OR Mozambique OR Namibia OR Niger OR Nigeria OR Rwanda OR “São Tomé and Príncipe” OR Senegal OR Seychelles OR Sierra Leone OR Somalia OR South Africa OR Sudan OR eSwatini OR Tanzania OR Togo OR Tunisia OR Uganda OR Zambia OR Zimbabwe) AND (sarcopen* OR muscl* atroph*) AND (prevalence)”. Then we adapted the search strategy for Web of Science and Embase.

For Embase, the following search strategy was used: “(sarcopenia/exp OR ‘sarcopenia’) AND (Africa/exp OR ‘Africa’ OR ‘Eastern Africa’ OR ‘Northern Africa’ OR ‘Southern Africa’ OR ‘Western Africa’ OR ‘africa, eastern’ OR ‘africa, northern’ OR ‘africa, southern’ OR ‘africa, western’ OR ‘east africa’ OR Angola/exp OR Algeria/exp OR Benin/exp OR Botswana/exp OR ‘Burkina Faso’/exp OR Burundi/exp OR Cameroon/exp OR ‘Cape Verde’/exp OR Chad/exp OR ‘Central African Republic’/exp OR Ciad/exp OR Comore/exp OR ‘Ivory Cost’/exp OR Congo/exp OR Egypt/exp OR Eritrea/exp OR Ethiopia/exp OR Gabon/exp OR Gambia/exp OR Ghana/exp OR Djibuti/exp OR Guinea/exp OR Kenya/exp OR Lesotho/exp OR Liberia/exp OR Libya/exp OR Madagascar/exp OR Malawi/exp OR Mali/exp OR Mauritania/exp OR Mauritius/exp OR Morocco/exp OR Mozambique/exp OR Namibia/exp OR Niger/exp OR Nigeria/exp OR Rwanda/exp OR ‘São Tomé and Príncipe’/exp OR Senegal/exp OR Seychelles/exp OR ‘Sierra Leone’/exp OR Somalia/exp OR ‘South Africa’/exp OR Sudan/exp OR eSwatini/exp OR Tanzania/exp OR Togo/exp OR Tunisia/exp OR Uganda/exp OR Zambia/exp OR Zimbabwe/exp). In Web of Science, we used the term sarcopenia (All Fields) with the names of the single African countries in all fields.

The management of potentially eligible references, at title/abstract level, was carried out using the Rayyan website (https://www.rayyan.ai/).

### Eligibility criteria

Inclusion criteria comprised the following: (1) observational studies (case–control, cohort, longitudinal studies); (2) studies that reported the diagnosis of sarcopenia according to all diagnostic criteria available including single (e.g., low physical performance) and multidimensional tools (e.g., European Working Group on Sarcopenia in Older People criteria) [9]; (3) studies carried out in Africa. In the cohort/longitudinal studies, data about prevalence were extracted. Only articles written in English were included. Studies focusing on specific medical conditions (e.g., cancer) or using screening tools for sarcopenia (e.g., SARC-F [19]) were excluded.

### Study selection

The selection of the articles was performed independently by two authors (NV, LS). One additional senior member (MV) of the review team was involved, when necessary. The study selection process involved, first, a selection based on title and/or abstracts, then a selection of studies retrieved from this first step based on the full-text manuscripts.

### Data collection and data items

We collected the following information: data regarding the identification of the manuscript (e.g., first author name and affiliation, year of publication, journal name, title of the manuscript), data on the characteristics of the population considered (e.g., sample size, mean age, country, gender, etc.), criteria used for defining sarcopenia, tools used for assessment of body composition, muscle strength and physical performance. These data were collected using a standard data extraction form in Microsoft Excel. The data extraction was carried out independently by one author (NV), with another author (MB) checking the quality of data extraction.

### Risk of bias evaluation

The Newcastle–Ottawa Scale (NOS) was used to assess the study quality/risk of bias [20]. The NOS assigns a maximum of 9 points based on three quality parameters: selection, comparability, and outcome. The evaluation was made by one author and checked by another, independently. The risk of bias was then categorized as high (< 5 points), moderate (6–7), or low (8–9) [21].

### Data synthesis

Cumulative prevalence and 95% confidence intervals (CIs) were estimated using a meta-analysis, under a random effect model [22]. Heterogeneity between estimates was assessed using the *I*^2^ statistic. In case of an *I*^2^ over 50%, a series of sensitivity analyses (criteria used for defining sarcopenia, tools used for assessment of body composition, muscle strength, and physical performance) were planned. However, only the first two analyses, i.e., criteria used for defining sarcopenia, tools used for assessment of body composition, were reported as only these reached a reliable number of studies. Publication bias was assessed by visually inspecting funnel plots and using Egger bias test, with a *p*-value < 0.05 indicative of possible publication bias [23]. In case of publication bias, the trim-and-fill analysis was performed [24]. All analyses were performed using “metaprop”, a command available in STATA 14.0.

## Results

### Literature search

**Fig. 1:**
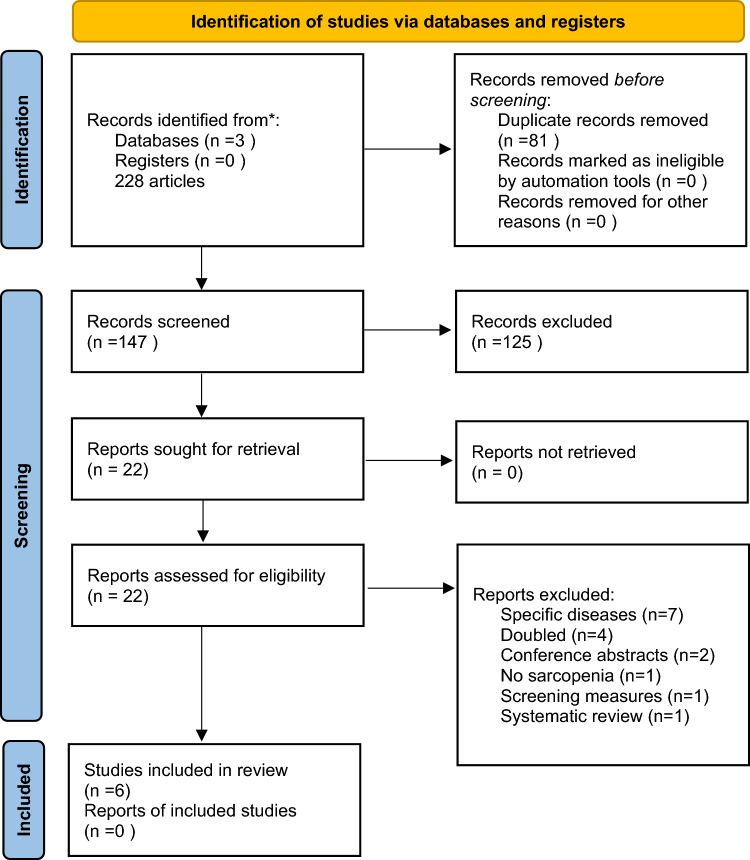
PRISMA flow-chart. (*From* [41]; For more information, visit: http://www.prisma-statement.org/)

As shown in Fig. 1, after removing duplicates, among 147 articles initially screened, we evaluated the full text of 22. Seven articles were excluded since they included people affected by a specific disease (such as cancer) (*n* = 7) or doubled, i.e., already included in one eligible study (*n* = 4) (the list of excluded references is provided in full in Supplementary Table 1). Of them, six articles, providing data for seven cohorts, reporting data regarding the prevalence of sarcopenia in Africa were included [25–30].

### Descriptive results and risk of bias

As shown in Table 1, the six articles [25–30] included a total of 10,656 participants across six different countries. The mean age of participants was 66.9 years (range: 56–74.1 years) and they were mainly female (58.1%). The identification of sarcopenia was performed mainly through criteria that integrated the evaluation of body composition, muscle strength, and/or physical performance parameters, such as those of the European Working Group on Sarcopenia in Older People criteria [9], while one study evaluated sarcopenia using only low muscle mass [30], and another only used poor physical performance [25]. The assessment of body composition was carried out using bioimpedance in two studies, DXA in two studies, or anthropometric measures in two studies. Muscle strength was assessed in all studies, except one in which muscle strength was not assessed [25]. Finally, physical performance was evaluated using usual gait speed in three studies (Table 1). The risk of bias was very low, indicating an excellent quality of the studies included (median NOS = 9) (Table 1).

**Table 1:** Descriptive characteristics of the participants included

Author, year	Country	Definition of sarcopenia	Assessment of body composition	Muscle strength	Physical performance	Sample size	Mean age	% of females	NOS
Alsadany, 2021	Egypt	EWGSOP2 criteria	BIA	Handgrip strength	Usual gait speed	127	68.7	48.9	9
Awotidebe, 2022	Nigeria	AWGS criteria, EWGSOP2 criteria	BIA	Handgrip strength	Usual gait speed	767	68.5	48.6	9
Jacob, 2023	Ghana	EWGSOP2 criteria	Anthropometric measures	Handgrip strength	Not reported	4892	72.8	55	9
Jacob, 2023	South Africa	EWGSOP2 criteria	Anthropometric measures	Handgrip strength	Not reported	3732	74.1	55	9
Metanmo, 2022	Cameroon	Only SPPB	Not reported	Not reported	SPPB	403	67.1	49.6	8
Ukegbu, 2018	South Africa	Only low muscle mass	DXA	Handgrip strength	Usual gait speed	247	56	100	8
Zengin, 2018	Gambia	EWGSOP criteria, FNIH criteria	DXA	Handgrip strength	Not reported	488	61	49.9	9
Total		Integrated criteria (*n* = 5), only low muscle mass (*n* = 1), only physical performance (*n* = 1)	BIA (*n* = 2), DXA (*n* = 2), anthropometric measures (*n* = 2), not measured (*n* = 1)	Handgrip strength (*n* = 6), not reported (*n* = 1)	Usual gait speed (*n* = 3), SPPB (*n* = 1), not reported (*n* = 3)	10,656	66.9	58.1	Median = 9

### Meta-analysis and sensitivity and meta-regression analyses

**Fig. 2:**
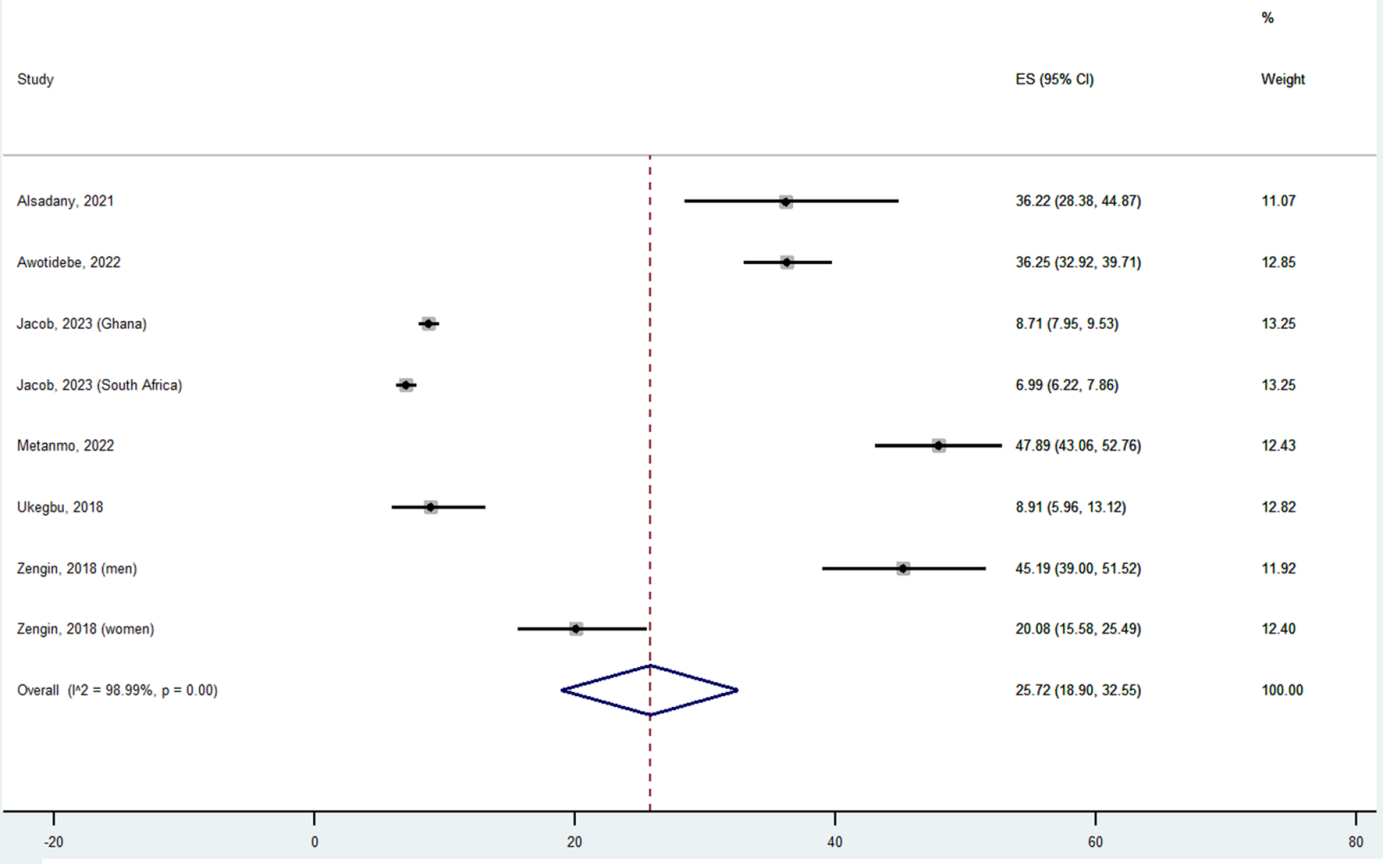
Prevalence of sarcopenia in Africa

Figure 2 shows the data regarding the meta-analysis of prevalence of sarcopenia in African countries. Overall, the weighted prevalence was 25.72% (95%CI: 18.90–32.55). This outcome was characterized by a high heterogeneity (*I*^2^ = 99%) as there was high variability in the prevalence with studies reported a prevalence of 6.99% [28] to 47.89% [25]. The outcome was characterized by publication bias (Egger’s test *p*-value = 0.01), although the trim-and-fill analysis did not change our results.

To explain heterogeneity found, several meta-regression and sensitivity analyses were ran. Regarding meta-regression analyses, higher mean age (beta = −0.004, *p* = 0.71) or higher presence of females (beta = −0.003, *p* = 0.13) did not moderate our results, indicating that these factors did not substantially affect our results. Supplementary Fig. 1 shows the data according to the criteria for defining sarcopenia. Overall, the study using only low muscle mass [30] for identifying sarcopenia reported a significantly lower prevalence of sarcopenia compared to criteria integrating several parameters (*p* for interaction < 0.0001). Similarly, Supplementary Fig. 2 shows that evaluating body composition using anthropometric measures led to a significantly lower prevalence of sarcopenia compared to BIA or DXA (*p* for interaction < 0.0001). Sensitivity analyses for muscle strength or physical performance tools were not conducted owing to a limited number of studies. Finally, when stratified by geographical area (Supplementary Fig. 3), we found the highest prevalence of sarcopenia in one study carried out in Central Africa (Cameroon) [25], and the lowest in two studies carried out in South Africa [28, 30].

## Discussion

In our systematic review with meta-analysis on the prevalence of sarcopenia across African countries, we found that approximately one in four people was categorized as sarcopenic.

Importantly, the prevalence of sarcopenia in Africa is very similar to other continents. For example, in a comprehensive systematic review of the literature including 263 studies, the authors found that the overall prevalence of sarcopenia ranged between 10% and 27% [13]. However, the existing research on sarcopenia in Africa is scarce, while 16% of the world’s population lives in Africa and many Africans may have poor access to good nutrition and health care [31]. Another important epidemiological point is that the prevalence of sarcopenia in Africa refers to a population having a mean age of 67 years, while sarcopenia is usually evaluated in people who are older (i.e., by 10 years or more) [13]. In our opinion, the prevalence found in our study indicates that sarcopenia could become a future emergency among African countries, and exploration of its role from a public health perspective is urgently needed in this continent.

Sarcopenia leads to a worse quality of life [32] and higher economic burden and health care cost [33]. Although sarcopenia has been recognized as a disease in the ICD for several years, the lack of a universal and standardized diagnostic criterion for sarcopenia remains one of the main issues for this condition [34]. In our opinion, the difficulty to make the diagnosis of sarcopenia homogeneous may also impact on the ability to produce uniform guidelines for the prevention and the treatment of this condition. As shown in this systematic review, the studies included used several approaches for the diagnosis of sarcopenia, from those integrating muscle strength, physical performance, and body composition parameters to those using only body composition, and this factor was a significant moderator of our analyses since the studies using a mono-dimensional definition reported significantly lower prevalence of sarcopenia. Therefore, our systematic review suggests that it is important to identify sarcopenia using several parameters. Another epidemiological finding of importance is that the prevalence of sarcopenia seems to be higher in Central Africa compared to South Africa. It may be argued that malnutrition is more prevalent in Central African countries than in South Africa, also among older people [35] and it is widely known that malnutrition is an important risk factor for sarcopenia [36].

In our view, the finding that sarcopenia affects one in four people living in Africa is of critical importance. It is widely known that sarcopenia is associated with several unfavorable outcomes in older people [37]. Moreover, it was preliminary reported that the consequences of sarcopenia could be more evident in people having less access to the health care system [38]. Therefore, future studies indicating whether the presence of sarcopenia in Africa could have a greater or a different role in prognosis are urgently needed.

The findings of our study must be interpreted within its limitations. First, we observed a large heterogeneity in our findings that, despite several meta-regression and sensitivity analyses, we were not able to explain. Second, the number of studies and participants was limited, particularly in comparison to other continents. However, we believe that our work may create an opportunity for further studies investigating the prevalence of sarcopenia in Africa. Finally, we were not able to explore as moderators of our findings some medical conditions, more frequent in Africa, such as infectious diseases that may affect our results. For example, it was reported by some investigations that the prevalence of sarcopenia in AIDS is extremely high [39] and AIDS is extremely common in this continent [40].

In conclusion, sarcopenia is a prevalent condition in Africa and thus research regarding this topic is a public health priority. Future studies that cover African countries for which data are not available and using standardized criteria are now needed.

### Supplementary Information

Below is the link to the electronic supplementary material.

Supplementary file1 (DOCX 2447 KB)

## References

[1] Cruz-Jentoft AJ, Sayer AA (2019). Sarcopenia. Lancet.

[2] Veronese N, Demurtas J, Soysal P (2019). Sarcopenia and health-related outcomes: an umbrella review of observational studies. Eur Geriatr Med.

[3] Cruz-Jentoft AJ, Landi F, Topinková E (2010). Understanding sarcopenia as a geriatric syndrome. Curr Opin Clin Nutr Metab Care.

[4] Anker SD, Morley JE, von Haehling S (2016) Welcome to the ICD‐10 code for sarcopenia. Wiley Online Library, pp 512–514 10.1002/jcsm.12147 PMC5114626 27891296

[5] Shafiee G, Keshtkar A, Soltani A (2017). Prevalence of sarcopenia in the world: a systematic review and meta-analysis of general population studies. J Diabetes Metab Disord.

[6] Damluji AA, Alfaraidhy M, AlHajri N (2023). Sarcopenia and cardiovascular diseases. Circulation.

[7] Catikkas NM, Bahat Z, Oren MM (2022). Older cancer patients receiving radiotherapy: a systematic review for the role of sarcopenia in treatment outcomes. Aging Clin Exp Res.

[8] Nishikawa H, Fukunishi S, Asai A (2021). Pathophysiology and mechanisms of primary sarcopenia. Int J Mol Med.

[9] Cruz-Jentoft AJ, Bahat G, Bauer J (2019). Sarcopenia: revised European consensus on definition and diagnosis. Age Ageing.

[10] Chen L-K, Liu L-K, Woo J (2014). Sarcopenia in Asia: consensus report of the Asian Working Group for Sarcopenia. J Am Med Dir Assoc.

[11] Fielding RA, Vellas B, Evans WJ (2011). Sarcopenia: an undiagnosed condition in older adults. Current consensus definition: prevalence, etiology, and consequences. International working group on sarcopenia. J Am Med Dir Assoc.

[12] Mayhew A, Amog K, Phillips S (2019). The prevalence of sarcopenia in community-dwelling older adults, an exploration of differences between studies and within definitions: a systematic review and meta-analyses. Age Ageing.

[13] Petermann-Rocha F, Balntzi V, Gray SR (2022). Global prevalence of sarcopenia and severe sarcopenia: a systematic review and meta-analysis. J Cachexia Sarcopenia Muscle.

[14] George-Carey R, Adeloye D, Chan KY (2012). An estimate of the prevalence of dementia in Africa: a systematic analysis. J Glob Health.

[15] Kalaria RN, Maestre GE, Arizaga R (2008). Alzheimer’s disease and vascular dementia in developing countries: prevalence, management, and risk factors. Lancet Neurol.

[16] Essomba MJN, Atsa D, Noah DZ (2020). Geriatric syndromes in an urban elderly population in Cameroon: a focus on disability, sarcopenia and cognitive impairment. Pan Afr Med J.

[17] Higgins JP, Thomas J, Chandler J (2019). Cochrane handbook for systematic reviews of interventions.

[18] Sarkis-Onofre R, Catalá-López F, Aromataris E (2021). How to properly use the PRISMA statement. Syst Rev.

[19] Malmstrom TK, Morley JE (2013). SARC-F: a simple questionnaire to rapidly diagnose sarcopenia. J Am Med Dir Assoc.

[20] Luchini C, Stubbs B, Solmi M (2017). Assessing the quality of studies in meta-analyses: advantages and limitations of the Newcastle Ottawa Scale. World J Meta-Anal.

[21] Luchini C, Veronese N, Nottegar A (2021). Assessing the quality of studies in meta-research: review/guidelines on the most important quality assessment tools. Pharm Stat.

[22] DerSimonian R, Laird N (1986). Meta-analysis in clinical trials. Control Clin Trials.

[23] Egger M, Smith GD, Schneider M (1997). Bias in meta-analysis detected by a simple, graphical test. BMJ.

[24] Duval S, Tweedie R (2000). A nonparametric “trim and fill” method of accounting for publication bias in meta-analysis. J Am Stat Assoc.

[25] Metanmo S, Kuate-Tegueu C, Gbessemehlan A (2022). Self-reported visual impairment and sarcopenia among older people in Cameroon. Sci Rep.

[26] Zengin A, Jarjou LM, Prentice A (2018). The prevalence of sarcopenia and relationships between muscle and bone in ageing West-African Gambian men and women. J Cachexia Sarcopenia Muscle.

[27] Awotidebe A, Bala A, Abdulkarim K (2022). Prevalence estimates of sarcopenia in community-dwelling older adults in Northern Nigeria according to revised European and Asian reference criteria. Physiother Q.

[28] Jacob L, Gyasi RM, Oh H (2023). Leisure-time physical activity and sarcopenia among older adults from low- and middle-income countries. J Cachexia Sarcopenia Muscle.

[29] Alsadany MA, Sanad HT, Elbanouby MH (2021). Detecting a valid screening method for sarcopenia in acute care setting. J Frailty Sarcopenia Falls.

[30] Ukegbu PO, Kruger HS, Meyer JD (2018). The association between calf circumference and appendicular skeletal muscle mass index of black urban women in Tlokwe City. J Endocrinol Metab Diabetes S Afr.

[31] Charlton KE, Rose D (2001) Nutrition among older adults in Africa: the situation at the beginning of the millenium. J Nutr 131:2424S–2428S 10.1093/jn/131.9.2424S 11533288

[32] Veronese N, Koyanagi A, Cereda E (2022). Sarcopenia reduces quality of life in the long-term: longitudinal analyses from the English longitudinal study of ageing. Eur Geriatr Med.

[33] Bruyère O, Beaudart C, Ethgen O (2019). The health economics burden of sarcopenia: a systematic review. Maturitas.

[34] Lee K, Shin Y, Huh J (2019). Recent issues on body composition imaging for sarcopenia evaluation. Korean J Radiol.

[35] Seid AM, Fentahun N (2022). Prevalence of malnutrition among old people in Africa: systematic review and meta-analysis. BMJ Open.

[36] Lengelé L, Bruyère O, Beaudart C (2021). Malnutrition, assessed by the Global Leadership Initiative on Malnutrition (GLIM) criteria but not by the mini nutritional assessment (MNA), predicts the incidence of sarcopenia over a 5-year period in the SarcoPhAge cohort. Aging Clin Exp Res.

[37] Beaudart C, Zaaria M, Pasleau F (2017). Health outcomes of sarcopenia: a systematic review and meta-analysis. PLoS ONE.

[38] Harris-Love MO, Adams B, Hernandez HJ et al (2014) Disparities in the consequences of sarcopenia: implications for African American Veterans. Frontiers Media SA, p 250 10.3389/fphys.2014.00250 PMC4083180 25071595

[39] SeyedAlinaghi S, Ghayomzadeh M, Mirzapour P (2023). A systematic review of sarcopenia prevalence and associated factors in people living with human immunodeficiency virus. J Cachexia Sarcopenia Muscle.

[40] Anabwani G, Navario P (2005). Nutrition and HIV/AIDS in sub-Saharan Africa: an overview. Nutrition.

[41] Page MJ, McKenzie JE, Bossuyt PM (2021). The PRISMA 2020 statement: an updated guideline for reporting systematic reviews. BMJ.

