# Supplementary material for: Prevalence of sarcopenia in Africa: a systematic review and meta-analysis of observational studies
Source: Aging Clin Exp Res. 2024 Jan 28;36(1):12. doi: 10.1007/s40520-023-02671-w (PMC10822818; doi:10.1007/s40520-023-02671-w)
Supplement: Supplementary file 1 — Supplementary file1 (DOCX 2447 KB) [file 40520_2023_2671_MOESM1_ESM.docx]

**Supplementary Table 1. Excluded references**

1. Leong DP, Teo KK, Rangarajan S, et al. (2016) Reference ranges of handgrip strength from 125,462 healthy adults in 21 countries: a prospective urban rural epidemiologic (PURE) study. J Cachexia Sarcopenia Muscle 7:535-546

2. Tyrovolas S, Koyanagi A, Olaya B, Ayuso-Mateos JL, Miret M, Chatterji S, Tobiasz-Adamczyk B, Koskinen S, Leonardi M, Haro JM (2016) Factors associated with skeletal muscle mass, sarcopenia, and sarcopenic obesity in older adults: a multi-continent study. J Cachexia Sarcopenia Muscle 7:312-321

3. Ngeuleu A, Allali F, Medrare L, Madhi A, Rkain H, Hajjaj-Hassouni N (2017) Sarcopenia in rheumatoid arthritis: prevalence, influence of disease activity and associated factors. Rheumatol Int 37:1015-1020

4. Golovach I, Zazirnyi I, Semeniv I (2018) How to assess comorbidity of the patients suffering from hip fracture associated with osteoporosis and subsequent hip replacement. Osteoporosis International 29:S199

5. Brennan-Olsen SL, Bowe SJ, Kowal P, Naidoo N, Quashie NT, Eick G, Agrawal S, D'Este C (2019) Functional Measures of Sarcopenia: Prevalence, and Associations with Functional Disability in 10,892 Adults Aged 65 Years and Over from Six Lower- and Middle-Income Countries. Calcif Tissue Int 105:609-618

6. Badran H, Elsabaawy MM, Ragab A, Aly RA, Alsebaey A, Sabry A (2020) Baseline Sarcopenia is Associated with Lack of Response to Therapy, Liver Decompensation and High Mortality in Hepatocellular Carcinoma Patients. Asian Pac J Cancer Prev 21:3285-3290

7. Mamphwe P, Kruger HS, Moss SJ, Cockeran M, Ricci C, Ukegbu PO, Kruger IM (2020) The association between anthropometric measures and physical performance in black adults of the North West Province, South Africa. Am J Hum Biol 32:e23324

8. Salman MA, Omar HSE, Mikhail HMS, et al. (2020) Sarcopenia increases 1-year mortality after surgical resection of hepatocellular carcinoma. ANZ J Surg 90:781-785

9. (2022) World Congress on Osteoporosis, Osteoarthritis and Musculoskeletal Diseases (WCO-IOF-ESCEO 2021): Poster Abstracts. Osteoporosis International 32:119-410

10. Gregson CL, Madanhire T, Rehman A, et al. (2022) Osteoporosis, Rather Than Sarcopenia, Is the Predominant Musculoskeletal Disease in a Rural South African Community Where Human Immunodeficiency Virus Prevalence Is High: A Cross-Sectional Study. J Bone Miner Res 37:244-255

11. Metelo-Liquito LD, Solomon C, Bhana-Nathoo D (2022) The prevalence of sarcopenia amongst non-small cell lung cancer patients, assessed using computed tomography, prior to treatment in a South African setting. SA Journal of Oncology 6:

12. Mohammed M, Li J (2022) Stroke-Related Sarcopenia among Two Different Developing Countries with Diverse Ethnic Backgrounds (Cross-National Study in Egypt and China). Healthcare (Basel) 10:

13. Smith L, Shin JI, Veronese N, et al. (2022) Sleep duration and sarcopenia in adults aged ≥ 65 years from low and middle-income countries. Aging Clinical and Experimental Research 34:1573-1581

14. El Miedany Y, El Gaafary M, Gadallah N, Mahran S, Fathi N, Abu Zaid MH, Tabra SAH, Hassan W, Elwakil W (2023) Osteoporosis treatment gap in patients at risk of fracture in Egypt: a multi-center, cross-sectional observational study. Arch Osteoporos 18:58

15. SeyedAlinaghi S, Ghayomzadeh M, Mirzapour P, et al. (2023) A systematic review of sarcopenia prevalence and associated factors in people living with human immunodeficiency virus. J Cachexia Sarcopenia Muscle 14:1168-1182

16. Zengin A, M OB, Parsons CM, Jarjou LM, Janha RE, Jobe M, Prentice A, Cooper C, Ebeling PR, Ward KA (2023) Sex-specific associations between cardiovascular risk factors and physical function: the Gambian Bone and Muscle Ageing Study. J Cachexia Sarcopenia Muscle 14:84-92

**Supplementary Figure 1. Prevalence of sarcopenia in Africa by criteria for definining sarcopenia**

**
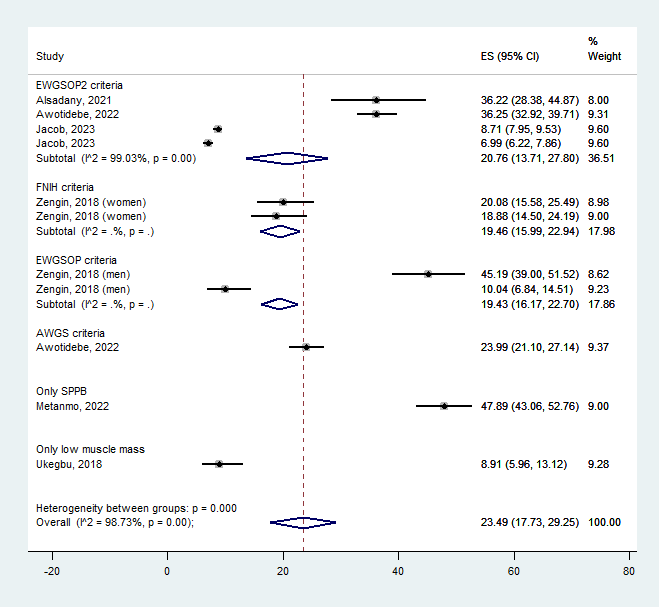
**

**Supplementary Figure 2. Prevalence of sarcopenia in Africa by type of body composition tool used**


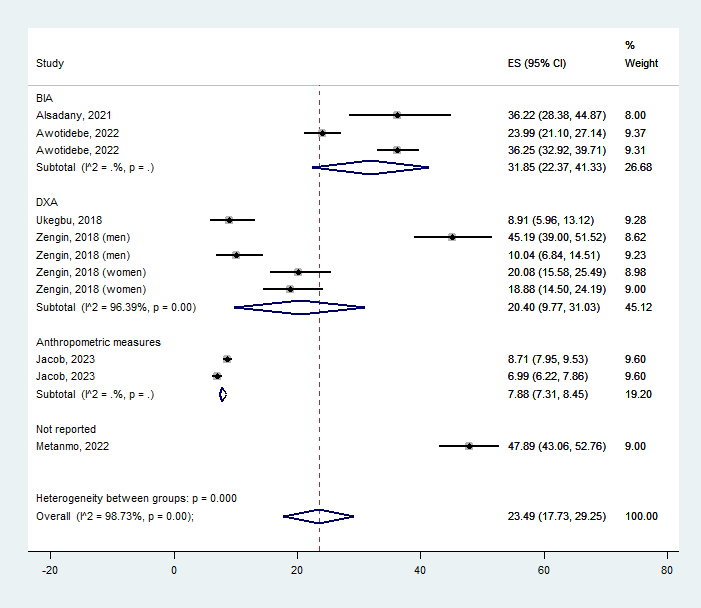


**Supplementary Figure 3. Prevalence of sarcopenia in Africa by geographical area**
